# Identification of Genetic Regions Associated with Scrotal Hernias in a Commercial Swine Herd

**DOI:** 10.3390/vetsci5010015

**Published:** 2018-01-27

**Authors:** Luisa Vitória Lago, Arthur Nery da Silva, Eraldo L. Zanella, Mariana Groke Marques, Jane O. Peixoto, Marcos V. G. B. da Silva, Mônica C. Ledur, Ricardo Zanella

**Affiliations:** 1 College of Veterinary Medicine, University of Passo Fundo, BR 285, São José, Passo Fundo, RS 99052-900, Brazil; lvitorialago96@gmail.com (L.V.L.); arthurnery97@gmail.com (A.N.S.); ezanella@upf.br (E.L.Z.); 2 Docentes do Programa de Pós-Graduação em Bioexperimentação-UPF, University of Passo Fundo, BR 285, São José, Passo Fundo, RS 99052-900, Brazil; 3 Embrapa Suínos e Aves, Rodovia BR-153, Km110, Concórdia, SC 89715-899, Brazil; mariana.marques@embrapa.br (M.G.M.); jane.peixoto@embrapa.br (J.O.P.); monica.ledur@embrapa.br (M.C.L.); 4 Embrapa Gado de Leite, Rua Eugênio do Nascimento, 610, Juiz de Fora, MG 36038-330, Brazil; marcos.vb.silva@embrapa.br

**Keywords:** swine, genetics, hernias, SNP

## Abstract

In this paper, we have used two approaches to detect genetic associations with scrotal hernias in commercial pigs. Firstly, we have investigated the effects of runs of homozygosity (ROH) with the appearance of scrotal hernias, followed by a Genome Wide Association Study (GWAS). The phenotype classification was based on visual appearance of scrotal hernias. Each affected animal was matched to a healthy control from the same pen. In the total, 68 animals were genotyped using the Porcine SNP60 Beadchip, out of those, 41 animals had the presence of hernias and 27 were healthy animals. Fifteen animals were removed from the analysis due to differences in genetic background, leaving 18 healthy animals and 35 piglets with scrotal hernia. Further, the detection of extended haplotypes shared ROH were conducted for health (control) and affected (case) animals and a permutation test was used to test whether the ROH segments were more frequent in case/case pairs than non-case/case pairs. Using the ROH, we have identified an association (*p* = 0.019) on chromosome 2(SSC2) being segregated on animals with the presence of scrotal hernias. Using a GWAS, a region composed by 3 SNPs on the sexual chromosome X (SSCX) were associated with scrotal hernias (*p* < 1.6 × 10^−5^), this region harbors the Androgen Receptor Gene (*AR*).

## 1. Introduction

Hernias are an abnormal protrusion of an organ or tissue through a defect, or natural opening, in the covering skin or muscle [1] and they are considered the most common congenital and developmental defect identified in the swine industry [2]. Affected animals will have a reduced feed efficiency and retarded growth and it is considered a cause of high morbidity and mortality [1,2,3]. As a result, piglets with hernias will have a lower sale value, causing economic losses to the producers, in addition to the animal effects as pain, stress and absence of welfare [2,3].

Hernias can be subdivided into scrotal or inguinal hernias and umbilical hernias [4]. The inguinal and scrotal hernias are not easily differentiated without clinical examination; therefore, they are generally classified as a single trait, even knowing that inguinal hernia is not sex limited trait [4]. Scrotal hernias are found at frequencies ranging from 1.7% to 6.7% [5]; however, they can exceed 7% depending on the pig breed, line and management practices [4]. The appearance of scrotal hernias is caused by the absence of obliteration of the process vaginalis after descent of the testis [6] or from a problem on the internal scrotal ring [1]. This allows the distal part of the jejunum and ileum to drop into the scrotum and it can rupture during castration. Nerveless, the intestines can migrate to the scrotum after castration.

It has been proposed that the appearance of scrotal hernias was linked to certain boar lines and families and the estimated heritability for this condition were moderate to high 0.2–0.86 [7,8,9]. However, even avoiding the use of those boar lines, hernias have not been completely eliminated from swine herds, indicating that the predisposing alleles to the formation of hernias are still segregating in the herds, possibly due to the maternal inheritance. In addition to that, several chromosomal regions on SSC1, SSC2, SSC5, SSC15, SSC 17 and SSCX have been identified to be associated with hernias, however not much progress have been acquired to eliminate this condition out of the herds [10,11,12]. Therefore, new approaches are needed to elucidate the genetic mechanisms involved with this condition.

The identification of genomic regions involved with the appearance of scrotal hernias is of great interest to the industry and to the producers, due to economic reasons and also to the animal welfare. Since hernias are being segregated in some animals from specific families’ lines, we have proposed that specific chromosomal segments (Haplotypes) are possibly carrying the alleles involved with this congenital defect.

Run of Homozygosity (ROH) is defined as two haplotypes carrying Identical by State (IBS) marker alleles from some position i through to some position j, where parents transmit those segments to the offspring [13,14,15]. In addition to that, the extent and frequency ROHs can infer about the ancestry and the breeding schemes of an individual in a population [13,14]. Generally, long segments ROH indicate recent inbreeding events and short segments of ROH may indicate older events of endogamic crosses in the population [13] and this increase in homozygosity levels may result in fixation of unfavorable alleles for a specific condition [15]. Therefore, in this study, we examined the effects of autosomal ROH with the appearance of scrotal hernias in swine and further conducted a genome wide association study to identify markers associated with the appearance of scrotal hernias in a cross bred swine population.

## 2. Material and Methods

This study was conducted at Embrapa Suínos e Aves, Santa Catarina State, Brazil and University of Passo Fundo, Rio Grande do Sul State, Brazil and had the approval of Institutional Animal Care and Use Committee for all experimental protocols used (CEUA/UPF 032/2014).

### 2.1. Experimental Population

Sixty-eight male piglets originated from a terminal cross of (Landrace x Large-White females and AGPIC 337 males) were used in this study. Animals had similar age and were from the same commercial swine herd located in the Northwest region of the Rio Grande do Sul State. Piglets were kept with the sow until 28 days of age and then weaned; castration was conducted during the first week of age. After weaning, piglets were moved to a group-housing with an average of 100 piglets per pen of mixed sex.

### 2.2. Phenotype Classification

The identification of affected animals, were visually conducted at 55 days of age. Each animal with scrotal/inguinal hernia was matched to a healthy control from the same pen.

### 2.3. Sample collection

Tissue samples were individually collected from animal´s ear (3 mm^2^). Samples were placed in regular micro centrifuge tubes and immediately refrigerated for further DNA extraction. The ear punching equipment used to collect the tissue samples was cleaned between each collection with bleach at 10% and 70% ethanol to avoid contamination among samples.

### 2.4. Genomic DNA Extraction and SNP Genotyping

Genomic DNA extraction was performed on the collected tissue samples, using PureLink^®^ Genomic DNA Mini Kit (Invitrogen- Carlsbad, CA, USA), according to the manufacturer instructions. The quantity and quality of DNA was measured using 260/280 nm wavelength readings using a NanoDrop ND-2000 spectrophotometer (NanoDrop Technologies Inc., Silverside, Wilmington, NC, USA). The 260/280 nm readings for all samples ranged between 1.8 and 2.0. DNA was diluted to 50 ng/μL in 10μL and genotyped at Deoxi Biotecnologia (Araçatuba, São Paulo, Brazil) with the Illumina PorcineSNP60V2 BeadChip (San Diego, CA, USA) whole-genome single nucleotide polymorphism (SNP) assay, which contains 61,565 SNPs distributed across the swine genome. Marker positions and distances were based on Sscrofa10.2, August 2011.

### 2.5. Data Quality Control

Data quality control was performed using PLINK V1.9 [16]. Animals were evaluated for the excess heterozygosity, to check for possible cross-contamination among samples. To test for differences in the genetic background of the animals, a multi-dimensional scaling (MDS) plot was constructed using PLINK [16] and the R statistical environment [17].

Most of the animals were partially related to each other with an average Identical by Decent (IBD) of 0.16 (0 to 0.55). After the removal of outlier animals, the levels of IBD increased to 0.24 (ranging from 0.07 to 0.53) indicating that all the animals in this study were related to each other.

The quality of SNPs was also assessed prior the analysis. SNPs were removed if they had a Minor Allele Frequency (MAF) < 1% or if they failed in more than 10% of the samples or if they fail Hardy Weinberg Equilibrium test at a *p*-value < 9.5×10^−7^. For the ROH segment sharing, additional SNP pruning was conducted, only autosomal markers, with a linkage disequilibrium (r^2^ < 0.2) were used in the analysis, due to differences in recombination in the sexual chromosomes [18]. PLINK was also used to compute the genomic inflation factor (ƛGC) and to construct a Quantile-Quantile Plot (Figure 2) to test for population substructure [16].

### 2.6. Genomic Inbreeding Estimation

Briefly, the runs of homozygosity were calculated with PLINK, using the following parameters: a minimum ROH of 100 SNPs with a minimum length of 1000 kb and one heterozygous SNP and one missing SNP genotype were allowed within a sliding window of 50 SNPs [16]. Identified ROHs were used to estimate individual genomic inbreeding coefficients (FROH) with the following formula [19]:

*FROH* = ∑_k_ Length (ROH_k_)/L

where “k” is the number of ROHs identified for each individual multiplied by the average length of its ROH segments in kilobases and “L” is the total swine genome length (2,808,525 kb, Sscrofa10.2, August 2011). A standard T-test was used to verify for differences in the inbreeding estimations between groups to evaluate the possible involvement of high levels of homozygosity and the incidence of scrotal hernias.

### 2.7. Segment Sharing Identification and Statistical Analysis

Shared Identical by Descent Segments were computed among all the animals for the identification of chromosomal shared regions using PLINK with a minimum segment length of 1000 kb and 100 SNPs per segment. Following, individuals from the same phenotype were then grouped together and the frequency of the appearance of shared segment were identified within the affected group (Case) and further compared with the frequency of the same shared segment found with non-affected animals (Control). Comparison within groups was conducted with all the animals. A permutation test with 100,000 permutations was used to calculate the existence of higher rate of case/case sharing segments than expected (Figure 4). For the segmental sharing regions, a significance was considered if *p* < 0.05.

The Genome-Wide Association Study (GWAS) was also conducted to identify loci associated with the presence of scrotal hernias. To test for the association between allele and the presence of scrotal hernias, a standard chi-square test and the odds ratio were computed using PLINK [16]. Significance was considered if *p* < 5 × 10^−5^ [20]. Further, the associated markers in the same chromosome were tested as a haplotype. The association test performed within PLINK has some limitations, as it considers all the makers in linkage equilibrium, therefore we have tested the LD among all of the markers in the same chromosome to evaluate if they could be considered part of the same region. Another limitation of PLINK was to calculate the additive effect of each marker. However, we used the logistic and beta functions to estimate the effect of each marker.

## 3. Results

**Figure 1:**
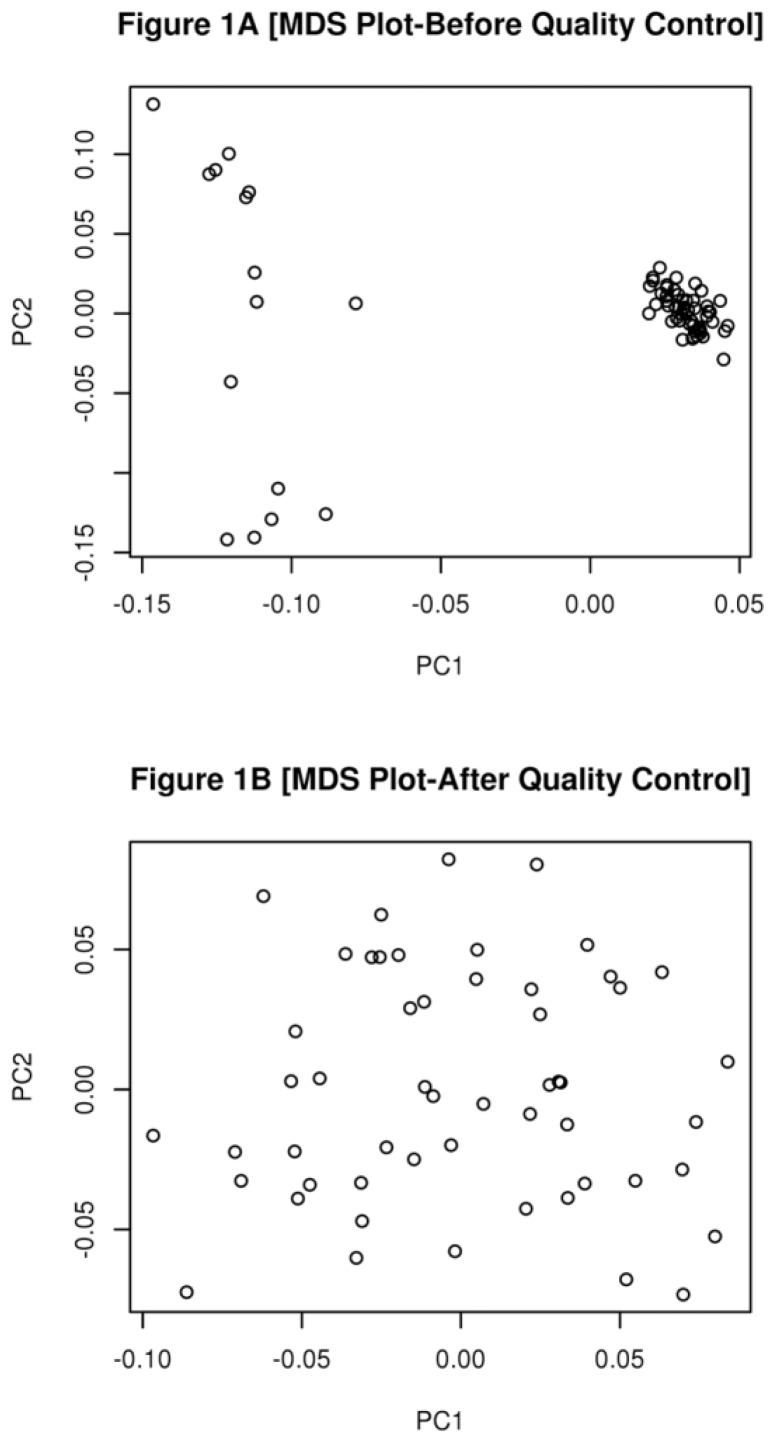
(**A**) A Multi-Dimensional scaling Plot constructed only with markers with LD < 0.2, before animal quality control. Each circle represents an individual animal; (**B**) A Multi-Dimensional Plot constructed only with markers with LD < 0.2, after animal quality control.

Since none of the samples had more than 10% of missing genotype or present evidence of cross contamination, they were only removed from the analysis due to differences in their genetic background. Fifteen samples (6 from animals with hernias and 9 from animals without hernias) were removed from the analysis, because they had differences in their genetic background, to avoid a spurious result (Figure 1A,B). 

**Figure 2:**
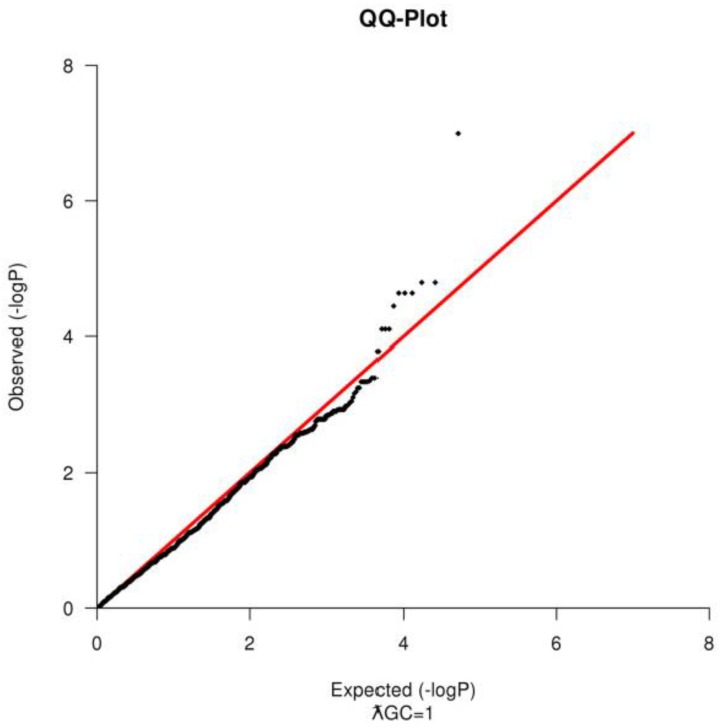
A Quantile-quantile Q-Q plot for observed *P*-values vs. those expected for an association of loci with scrotal hernias formation. The Q-Q plot shows no evidence of population substructure.

Based on the SNP quality control, 1456 markers failed in more than 10% of the samples, 6876 had a MAF < 1% and 2436 failed in both categories; therefore, they were excluded from the analysis. For the Hardy Weinberg Equilibrium Test, 393 SNPs were additionally removed. After sample and SNP quality control, 50,797 SNPs and 53 animals (18 healthy animals and 35 piglets with scrotal hernia) remained to be used in the association test. There was no evidence of population stratification when tested using the QQ-Plot (Figure 2) and the ƛGC = 1, after the removal of the outliers. For the Shared Identical by Descent Segments among animals, only *n* = 3621 SNPs with a *r^2^* < 0.2 were remained (Table 1).

There were no differences in the genomic inbreeding estimation between the animals with hernias (FROH = 0.037) and the healthy animals (FROH = 0.034) *p* > 0.29). The average inbreeding between all the animals in this study was FROH = 0.036 (ranging from 0.017 to 0.067), indicating low levels of homozygosity among the animals used from this population.

**Figure 3:**
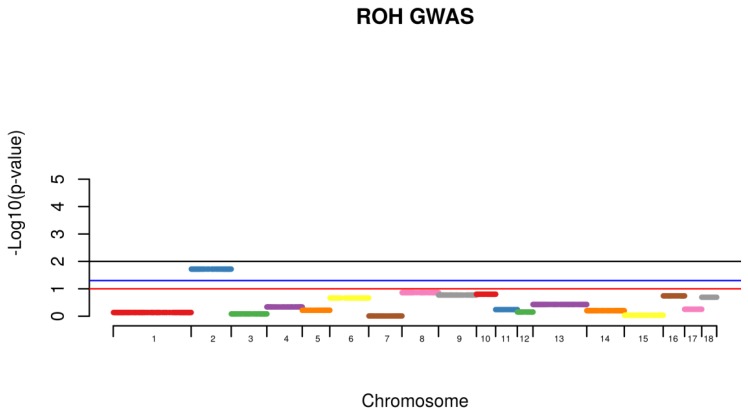
ROH segment sharing permutation test with the appearance of scrotal hernias in a commercial swine operation, constructed with 100 SNPs and 1000 Kb distance.

Using the IBD segmental sharing among animals, a signal was identified to be segregating among animals with hernias on SSC2 (Figure 3).

**Figure 4:**
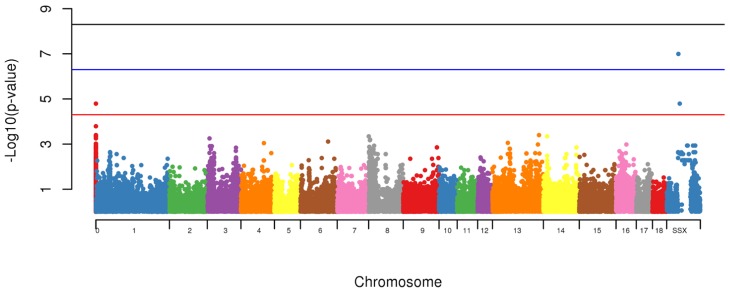
Case and Control association test with appearance of scrotal hernias with a sample population from a commercial swine operation.

Using the GWAS, two markers (MARC0114274, *p* = 1.6 × 10^−7^ and MARC0063079 (*p* < 1.6 × 10^−5^), located on SSCX at 104,313,388 bp and 115,135,321 bp, respectively and one with unknown location based on Sscrofa10.2 (CASI0004285) however using the Sscrofa11.1 built it was located on SSCX 53,940,129 bp (*p* < 1.6 × 10^−5^) were associated with appearance of scrotal hernias in this population (Figure 4).

## 4. Discussion

Hernias is considered a major health problem being consistently identified in different species, therefore the identification of genetic mechanisms and genes being identified with this condition will elucidate possible treatments and prevention. Regions of Homozygosity, also known as ROH are used as an accurate estimation of inbreeding levels and also to predict the period when the endogamic crosses have occurred. Long stretches of ROH are generally related to newly events of inbreeding, whereas short stretches are associated with older events. In addition to that, higher levels of homozygosity can increase the chances of deleterious alleles to be expressed in populations [13,14,19]. The identification of shared regions was conducted for healthy (control) and affected animals (case) and a permutation test was used to verify whether the IBD segments were more frequent in case/case pairs than control/case or control/control pairs. A signal was identified to be segregated among animals with hernias on SSC2 (Figure 4). This chromosome was identified to harbor a locus associated with susceptibility to scrotal/inguinal hernias, using a genome-wide scan in a White Duroc × Erhualian cross [10]. Grindflek and colleagues [11] using a family based test (TDT), have similar findings on SSC2. A region on SSC2 was previously identified to be linked with scrotal hernias using an affected sib pair test [11]. However, in this study, we used the ROH analysis related to the onset of scrotal hernia, unlike the other studies cited. One of the limitations of the use of this technique was the limited number of markers in low linkage disequilibrium found per chromosome in this population. On SSC2, the chromosome that presented the highest signal for the presence of ROH segment sharing being segregated with affected animals, only 225 SNPs were used to construct the sharing segments. However, this methodology is very powerful to detect segment regions being segregated among affected animals, using a small sample size. The LD between the two associated markers on SSCX (MARC0114274 and CASI0004285) was *r^2^* = 0.216, indicating a possible independent involvement of each marker. The LD between the markers on SSCX and the marker with unknown position was *r^2^* < 0.3, indicating that the markers are possibly being segregated independently from each other.

When the combined effect of the markers MARC0114274 and CASI0004285 were tested, it improved the significance of the association to *p* = 2.1 × 10^−8^, being suggestive an additive effect of markers to the appearance of hernias. The haplotype composed of markers (TA) was found in 33% of the control animals and it was never observed in the affected group (Table 2). However, the haplotype (GC) was identified in 82.86% of the affected animals and in 38% of control group. Possibly the alleles TA are conferring to the animals less susceptibility to the predisposition to the formation of scrotal hernias in this swine population.

Testing for the combined effect of markers MARC0114274 and MARC0063079 improved the association to *p* = 1.41 × 10^−9^ and the haplotype composed of TA was identified in 44.4% of control animals and it was never observed in the affected piglets. The opposite haplotype (GG) was identified in 82.86% of the affected animals and in 44.4% of control group. The allele T for the SNP MARC0114274 was constantly being identified in healthy animals and their combined effect with the allele A of the MARC0063079 SNPs was never identified in affected individuals.

When evaluating the combined effect of markers CASI0004285 and MARC0063079, it also improved the significance of the association test to *p* = 2.90 × 10^−7^. The haplotype AA was found in 33% of the control animals and never found in the affected group. These results are congruent with the results of the combined effect of the associated markers. When we tested the effect of all three markers together (MARC0114274, CASI0004285 and MARC0063079), the association was *p* = 9.5 × 10^−8^, with 33% of the control animals having the haplotype (TAA) and none of the affected group presenting this haplotype. The haplotype GCG was found in 74.29% of the affected group and in 38.89% of the control group. These support our assumption of the individual SNP effect to contribute to the appearance of scrotal hernias in a commercial swine operation. Possibly those markers are part of a QTL which harbor an important gene involved with the predisposition to hernias.

Interestingly, this region (53,940,129 bp–115,135,321 bp) on chromosome X harbors the Androgen Receptor gene (*AR*; chrX:60,314,522 bp–60,504,529 bp Sscrofa10.2), which is a great candidate gene for the inguinal hernias, trait that is under our study. Mutations in the androgen receptor that causes androgen insensitivity have been previously associated with inguinal hernia [21].

Most of the inguinal and scrotal hernias are associated with birth defects in human, under the control of genetic and environmental factors. However, in the swine industry, the incidence of inguinal and scrotal hernias can vary, depending on the pig breed and lines being used, therefore it is believed that genetic factors have an enormous effect on hernia development [9]. Previous studies using microsatellite data have detected 9 significant QTL on 8 porcine chromosomes in a population using affected sib pairs through a genome-wide linkage analysis including the SSCX in pigs [11]. Other studies have identified and confirmed QTLs on SSC2 and SSC12 to be associated with scrotal hernias in pigs [22]. Therefore, it is proposed that hernias are a polygenic condition with the involvement of several candidate genes, which our data support this information. Some of the candidate genes involved with this condition include insulin-like 3 (INSL3) [23], beta-glucuronidase gene (GUSB) [24] and the SRY-box 9 (SOX9) gene [10]. Even knowing that those genes are not near our identified regions, it is possible that those markers are involved with predisposition of scrotal hernias. The existence of individual and haplotype-specific differences in recombination rates on SSCX, makes it very difficult to narrow QTL interval using traditional fine-mapping approaches in pigs, which may have explained the absence of genes near the associated markers [18]. Sawaguchi and colleagues have suggested that the genetic mode inheritance of scrotal hernias is a sex-limited autosomal trait with partial penetrance [25,26]. However, in our study it was observed that the sexual chromosome has an important role in the appearance of this trait in pigs.

## 5. Conclusions 

Our study was able to identify a signal on SSC2 using a ROH segment sharing region and three individual markers using a GWAS, all of then being located in the sexual chromosome near the Androgen Receptor gene, being involved with the appearance of scrotal hernias in a commercial swine operation. In addition, no association was observed between the levels of homozygosity and the appearance of scrotal hernias in pigs.

## Figures and Tables

**Table 1:** Number of markers per chromosome used for the identification of ROH shared segments for the permutation test among case and control animals.

Chromosome	Markers
1	353
2	225
3	196
4	238
5	190
6	181
7	257
8	230
9	202
10	175
11	162
12	132
13	259
14	235
15	238
16	130
17	121
18	97
Total	3621

**Table 2:** Haplotypes constructed with associated markers, with the frequency being identified in Cases and Control animals.

Marker	Haplotype	% Case	% Control
MARC0114274-CASI0004285	TA	0.00%	33.00%
	GC	82.86%	38.00%
MARC0114274-MARC0063079	TA	0.00%	44.4%
	GG	82.86%	44.4%
CASI0004285-MARC0063079	AA	0.00%	33.00%
MARC0114274-CASI0004285-MARC0063079	TAA	0.00%	33.00%
	GCG	74.29%	38.29%
